# Nutritional trends among children under the age of five in Zambia: A repeated cross sectional analysis using Zambia demographic health survey (2002–2018)

**DOI:** 10.1371/journal.pone.0329998

**Published:** 2026-02-25

**Authors:** Namukolo Mukubesa, Leah Kamulaza, Mutale Sampa, Atupele Chisiza, Nasilele Amatende, Hope Sabao, Wilbroad Mutale

**Affiliations:** 1 Southern African Institute for Collaborative Research and Innovation Organization (SAICRIO), Lusaka, Zambia; 2 The University of Zambia, School of Natural and Applied Sciences, Department of Mathematics, Statistics and Actuarial Science; 3 The University of Zambia, School of Public Health, Department of Epidemiology and Biostatistics, Lusaka, Zambia; 4 University of Witwatersrand, Division of Epidemiology and Biostatistics; 5 The University of Zambia, School of Public Health, Department of Health Policy and Systems, Lusaka, Zambia; Bangladesh Institute of Social Research (BISR) Trust, BANGLADESH

## Abstract

Childhood undernutrition, manifested as stunting, wasting, and underweight, remains a major public health challenge, particularly in low- and middle-income countries. In Zambia, the burden of undernutrition remains persistently high despite ongoing interventions. This study analyzed trends and determinants of nutritional status among children under five years using data from the Zambia Demographic and Health Survey (ZDHS) conducted in 2002, 2007, 2013–14, and 2018. The analysis assessed the prevalence of stunting, underweight, and wasting in children aged 0–59 months, using mean Z-scores and standard deviations. Logistic regression models were applied to identify key socio-demographic and health-related risk factors, with analyses performed in Python, accounting for survey design and weights. Findings revealed a notable decline in malnutrition: stunting dropped from 45.6% in 2002 to 34.7% in 2018; underweight from 27.3% to 11.6%; while wasting remained stable at approximately 4%. Severe stunting and underweight also decreased significantly, whereas severe wasting fluctuated. Prevalence rates were higher when excluding children under six months (left-truncated data), suggesting possible protection from early infancy due to exclusive breastfeeding. Key predictors of malnutrition included low birth weight, poverty, regional disparities, and diarrheal episodes. Despite progress, stunting remains a pressing concern. The higher rates observed in older infants point to the need for strengthened interventions targeting the postnatal period. Enhancing maternal and child health services, improving nutrition programs, and addressing poverty are critical to sustaining reductions in childhood malnutrition.

## 1. Introduction

Malnutrition in early childhood remains a major public health concern, shaping long-term health outcomes and economic productivity at both individual and national levels [1,2]. This issue is particularly pressing in Africa, where child undernutrition stands as one of the continent’s fundamental challenges to improved development, significantly slowing progress toward the goal of reducing malnutrition [3]. Millions of children worldwide experience the devastating effects of chronic and acute undernutrition, which not only increases the risk of mortality but also impairs cognitive development and physical growth. The three key indicators of undernutrition which are stunting, wasting, and underweight reflect different dimensions of nutritional deficits. Stunting results from prolonged nutritional deprivation, underweight signals, recent severe weight loss, and wasting encompasses aspects of both Ministry of Health (MOH) [4,5]. Children whose height-for-age Z-score falls below minus two standard deviations (−2 SD) from the median of the reference population are classified as stunted.

Wasting, on the other hand, is characterized by a swift loss of body weight and muscle tissue, resulting in a low weight-for-height. Like stunting, it is evaluated through a Z-score comparison, with children falling below minus two standard deviations (−2 SD) considered wasted. Underweight, a broader term, indicates a child has a low weight-for-age. Similar to the other indicators, underweight is assessed through a Z-score comparison, with children below minus two standard deviations (−2 SD) classified as underweight.

The prevalence of stunting is decreasing, while the prevalence of overweight is increasing worldwide [6]. In developing countries, approximately 32.0% of children under five years old are stunted, and 10.0% are wasted [5]. In 2014, 57.0% and 37.0%, 68.0%, and 28.0%, and 48.0% and 25.0% of all stunted, wasted, and overweight children under five lived in Asia and Africa, respectively [6]. Malnutrition is responsible for at least 35.0% of deaths among children under five worldwide [7], and nearly half of under-five deaths can be attributed to undernutrition [8].

In Zambia, despite sustained efforts to address child malnutrition, progress has been uneven. While stunting has declined moderately over the past decades, wasting and underweight prevalence rates have shown inconsistent trends, indicating persistent nutritional challenges. Similar patterns have been observed in other sub-Saharan African countries. For instance, a multi-country analysis by [9] found that while stunting has declined in some regions, wasting and underweight remain prevalent due to food insecurity, poor maternal education, and inadequate health services [9]. Existing research often focuses on cross-sectional estimates of malnutrition without addressing potential biases introduced by left truncation, a methodological issue where children younger than six months, regardless of survival status, are excluded from analysis.

This study employs data from the Zambia Demographic and Health Surveys (ZDHS) spanning 2002–2018 to examine long-term trends and risk factors of malnutrition. By comparing complete datasets (0–59 months) with left-truncated datasets (6–59 months), we assess how exclusion of the youngest age group influences prevalence estimates and statistical associations. Our analysis suggests that malnutrition rates appear slightly higher in left-truncated data, reinforcing the need to account for early-life vulnerabilities in nutritional assessments. Additionally, logistic regression models identify key risk factors, providing insights into socioeconomic and demographic determinants of malnutrition.

A more comprehensive understanding of malnutrition dynamics, particularly in early infancy, is crucial for designing targeted interventions. This study highlights the importance of strengthening early nutritional programs, refining assessment methodologies, and addressing gaps in policy to mitigate the long-term consequences of childhood malnutrition in Zambia.

## 2. Materials and methods

### 2.1. Dataset and study design

This study utilized nationally representative data on malnutrition from the ZDHS for the years 2002, 2007, 2013–14, and 2018. The ZDHS is a comprehensive household survey conducted periodically to collect data on health and demographic indicators, including child nutrition. These datasets provide critical insights into malnutrition trends among children under the age of five in Zambia.

For this analysis, we focused on the child’s recode file, which contains detailed information on child health, nutritional status, and socio-demographic characteristics. The initial sample sizes for the respective survey years were 6,031 (2002), 5,951 (2007), 12,040 (2013–14), and 9,959 (2018). After excluding cases with missing values on key anthropometric and demographic variables using list wise deletion the final analytical samples were reduced to 5,598 (2002), 5,351 (2007), 11,555 (2013–14), and 8,808 (2018).

The study employed a repeated cross sectional approach to examine trends and determinants of malnutrition over time. Descriptive statistics, trend analyses, and regression models were used to assess changes in malnutrition prevalence and identify key determinants influencing child nutritional outcomes over the study period.

### 2.2. Ethical considerations

This study utilized retrospective, publicly available data from ZDHS conducted in 2002, 2007, 2013–14, and 2018. All surveys received ethical approval from the relevant national ethical review committees in Zambia and from the Institutional Review Board of ICF. Access to the anonymized datasets was granted by The DHS Program upon request. Data for this study were downloaded and accessed for analysis between March 10 and March 15, 2024. The authors did not have access to any personally identifiable information at any stage of the analysis.

### 2.3. Response variable and explanatory variables

In this study, we examined three types of response variables: stunting, underweight and wasting. These variables were assessed using height-for-age Z-score (HAZ), weight-for-height Z-score (WHZ), and weight-for-age Z-score (WAZ). Z-scores were calculated utilizing World Health Organization (WHO) AnthroPlus, with age, weight, and height as determinants. Stunting was identified if HAZ fell below −2 standard deviations (SD), while wasting and underweight were characterized by WHZ and WAZ below −2 SD, respectively [10].

Various socio-economic and demographic factors were selected as explanatory variables, drawing from existing literature. Detailed descriptions of these variables, along with their respective categories, are presented in Tables below.

### 2.4. Statistical analysis

#### Identification of determinant of undernutrition.

To identify potential determinants of undernutrition, preliminary descriptive analyses were conducted using chi-square tests for categorical variables and independent t-tests for continuous variables to examine differences between affected and non-affected groups. The results of these analyses are presented in Tables 1–3, which summarize the baseline and socio-demographic characteristics and their associations according to stunting, underweight, and wasting status. These bivariate analyses were used to describe patterns in the data and to inform the multivariable logistic regression models, alongside evidence from prior literature and biological plausibility.

**Table 1:** Baseline and demographic characteristic of respondents for stunting and its associations.

Variables	Stunting		P-value
	No	Yes	
**Respondent age group** ≤24 25-34 ≥35	6066 (59.5%) 8810 (61.5%) 4162 (61.9%)	4129 (40.5%) 5513 (38.5%) 2564 (38.1%)	<0.05
**Region** Central C/belt Eastern Luapula Lusaka Muchinga N/Western Northern Southern Western	1154 (63.4%) 1223 (67.1%) 1533 (63.0%) 1348 (58.2%) 1304 (66.5%) 1202 (62.2%) 1274 (66.1%) 1233 (54.4%) 1473 (68.0%) 1130 (68.4%)	667 (36.6%) 600 (32.9%) 901 (37.0%) 970 (41.8%) 656 (33.5%) 731 (37.8%) 653 (33.9%) 1033 (45.6%) 693 (32.0%) 522 (31.6%)	<0.01
**Size of child** Average large small	11370 (60.7%) 5666 (65.8%) 2002 (51.3%)	7358 (39.3%) 2944 (34.2%) 1904 (48.7%)	<0.01
**Wealth Index** Middle Poor Rich	3484 (62.5%) 7293 (58.1%) 5213 (69.3%)	2092 (37.5%) 5256 (41.9%) 2310 (30.7%)	<0.01
**Region** Rural Urban	12517 (58.6%) 6521 (66.0%)	8842 (41.4%) 8842 (41.4%)	<0.01
**Education Level** Tertiary No education Primary Secondary	786 (83.4%) 2172 (57.2%) 10479 (58.0%) 5601 (66.3%)	156 (16.6%) 1623 (42.8%) 7574 (42.0%) 2853 (33.7%)	<0.01
**Mother work status** No Yes	8865 (62.2%) 10173 (59.9%)	5389 (37.8%) 6817 (40.1%)	<0.01
**Gender** Female Male	9909 (63.3%) 9129 (58.6%)	5746 (36.7%) 6460 (41.4%)	<0.01
**Toilet facility** Other Pit latrine Water closet	5329 (56.8%) 10774 (61.6%) 2935 (67.1%)	4049 (43.2%) 6719 (38.4%) 1438 (32.9%)	<0.01
**Breastfeeding status** No Yes	7751 (59.0%) 11287 (62.3%)	5388 (41.0%) 6818 (37.7%)	<0.01
**Water source** Borehole Other Tap Well	3137 (63.3%) 475 (62.9%) 50 23 (66.9%) 10403 (57.7%)	1817 (36.7%) 280 (37.1%) 2489 (33.1%) 7620 (42.3%)	<0.01
**Diarrhea** No Yes	15946 (61.7%) 3092 (57.5%)	9918 (38.3%) 2288 (42.5%)	<0.01
**Fever** No Yes	14650 (61.9%) 4388 (58.1%)	9035 (38.1%) 3171 (41.9%)	<0.01
**Cough** No Yes	14098 (61.0%) 4940 (60.8%)	9019 (39.0%) 3187 (39.2%)	0.76
**Age first pregnancy** **in years**	Mean(SD)	Mean(SD)	<0.01
	18.5 (0.02)	18.2 (0.03)	

**Table 2:** Baseline and demographic characteristic of respondents for underweight and its associations.

Variables	Underweight		P-value
	No	Yes	
**Respondent age group** ≤ 24 25-34 ≥ 35	8530 (83.7%) 12079 (84.3%) 5676 (84.4%)	1665 (16.3%) 2244 (15.7%) 1050 (15.6%)	0.3
**Province** Central Copperbelt Eastern Luapula Lusaka Muchinga N/Western Northern Southern Western	1594 (87.5%) 1580 (86.7%) 2188 (89.9%) 1914 (82.6%) 1753 (89.4%) 1642 (84.9%) 1687 (87.5%) 1898 (83.8%) 1926 (88.9%) 1405 (85.0%)	227 (12.5%) 243 (13.3%) 246 (10.1%) 404 (17.4%) 207 (10.6%) 291 (15.1%) 240 (12.5%) 368 (16.2%) 240 (11.1%) 247 (15.0%)	<0.01
**Size of child** Average large small	15733 (84.0%) 7637 (88.7%) 2915 (74.6%)	2995 (16.0%) 973 (11.3%) 991 (25.4%)	<0.01
**Wealth Index** Middle Poor Rich	4893 (87.8%) 10564 (84.2%) 6758 (89.8%)	683 (12.2%) 1985 (15.8%) 765 (10.2%)	<0.01
**Region** Rural Urban	17731 (83.0%) 8554 (86.5%)	3 628 (17.0%) 1331 (13.5%)	<0.05
**Education Level** Tertiary No Education Primary Secondary	878 (93. 2%) 3025 (79.7%) 14991 (83.0%) 7391 (87.4%)	64 (6.8%) 770 (20.3%) 3062 (17.0%) 1063 (12.6%)	<0.01
**Mother work status** No Yes	12075 (84.7%) 14210 (83.6%)	2179 (15.3%) 2780 (16.4%)	<0.05
**Gender** Female Male	13363 (85.4%) 12922 (82.9%)	2292 (14.6%) 2667 (17.1%)	<0.05
**Toilet facility** Other Pit latrine Water closet	7419 (79.1%) 15113 (86.4%) 3753 (85.8%)	1959 (20.9%) 2380 (13.6%) 620 (14.2%)	<0.05
**Breastfeeding status** No Yes	11102 (84.5%) 15183 (83.9%)	2037 (15.5%) 2922 (16.1%)	0.135
**Water source** Borehole Other Tap Well	4303 (86.9%) 636 (84.2%) 6508 (86.6%) 14838 (82.3%)	651 (13.1%) 119 (15.8%) 1004 (13.4%) 3185 (17.7%)	<0.05
**Diarrhea** No Yes	22041 (85.2%) 4244 (78.9%)	3823 (14.8%) 1136 (21.1%)	<0.05
**Fever** No Yes	20313 (85.8%) 5972 (79.0%)	3372 (14.2%) 1587 (21.0%)	<0.05
**Cough** No Yes	19611 (84.8%) 6674 (82.1%)	3506 (15.2%) 1453 (17.9%)	<0.05
**Age first pregnancy** **in years**	Mean (SD)	Mean (SD)	<0.01
	18.5 (2.96)	18.3 (2.86)	

**Table 3:** Baseline and demographic characteristic of respondents for wasting and its associations.

Variables	Wasting		P-value
	No	Yes	
**Respondent age group** ≤ 24 25-34 ≥ 35	9662 (94.8%) 13606 (95.0%) 6362 (94.6%)	533 (5.2%) 717 (5.0%) 364 (5.4%)	0.43
**Province** Central C/belt Eastern Luapula Lusaka Muchinga N/Western Northern Southern Western	1742 (95.7%) 1731 (95.0%) 2315 (95.1%) 2115 (91.2%) 1837 (93.7%) 1823 (94.3%) 1824 (94.7%) 2197 (97.0%) 2090 (96.5%) 1568 (94.9%)	79 (4.3%) 92 (5.0%) 119 (4.9%) 203 (8.8%) 123 (6.3%) 110 (5.7%) 103 (5.3%) 69 (3.0%) 76 (3.5%) 84 (5.1%)	<0.01
**Size of child** Average large small	17765 (95.0%) 8180 (95.2%) 3644 (93.3%)	938 (5.0%) 414 (4.8%) 260 (6.7%)	<0.05
**Wealth Index** Middle Poor Rich	5324 (95.5%) 11851 (94.4%) 7118 (94.6%)	252 (4.5%) 698 (5.6%) 405 (5.4%)	<0.05
**Region** Rural Urban	20272 (94.9%) 9358 (94.7%)	1087 (5.1%) 527 (5.3%)	0.38
**Education Level** Tertiary No Education Primary Secondary	896 (95.1%) 3566 (94.0%) 17155 (95.0%) 8013 (94.8%)	46 (4.9%) 229 (6.0%) 898 (5.0%) 441 (5.2%)	0.8
**Mother work status** No Yes	13527 (94.9%) 16103 (94.8%)	727 (5.1%) 887 (5.2%)	0.65
**Gender** Female Male	14919 (95.3%) 14711 (94.4%)	736 (4.7%) 878 (5.6%)	<0.05
**Toilet facility** Other Pit latrine Water closet	6194 (95.0%) 16143 (94.7%) 3539 (94.7%)	323 (5.0%) 903 (5.3%) 200 (5.3%)	0.39
**Breastfeeding status** No Yes	12544 (95.5%) 17086 (94.4%)	595 (4.5%) 1019 (5.6%)	<0.05
**Water source** Borehole Other Tap Well	4706 (95.1%) 650 (95.0%) 6059 (94.5%) 14374 (94.8%)	244 (4.9%) 34 (5.0%) 355 (5.5%) 785 (5.2%)	0.69
**Diarrhea** No Yes	24608 (95.1%) 5022 (93.3%)	1256 (4.9%) 358 (6.7%)	<0.05
**Fever** No Yes	22484 (94.9%) 7146 (94.5%)	1201 (5.1%) 413 (5.5%)	0.18
**Cough** No Yes	21909 (94.8%) 7721 (95.0%)	1208 (5.2%) 406 (5.0%)	0.44
**Age first pregnancy** **in years**	Mean (SD)	Mean (SD)	0.42
	18.5 (2.92)	18.5 (3.01)	

Subsequently, logistic regression analyses were performed to assess associations between potential risk factors and the three undernutrition indicators (stunting, wasting, and underweight). To account for the complex DHS survey design, all regression models incorporated sampling weights and clustering at the primary sampling unit (PSU) level. Both unadjusted and adjusted odds ratios (ORs and AORs) with 95% confidence intervals (CIs) were estimated, and the results are presented in Tables 7–9. All analyses were conducted using Python with appropriate libraries for survey-adjusted regression.

**Table 4:** Trends of anthropometric indicators in children aged 0 to 59 months (2002 to 2018) Zambia.

Variable	2001 (n = 5598)		2007 (n = 5351)		2013 (n = 11555)		2018 (n = 8808)	
	Mean (SD)	%	Mean (SD)	%	Mean (SD)	%	Mean (SD)	%
Stunting	−1.89 (1.5)	45.6	−1.65 (1.7)	42.2	−1.56 (1.16)	38.1	−1.48 (1.43)	34.7
Underweight	−1.31 (1.2)	27.3	−0.81 (1.14)	13.5	−0.9 (1.11)	14.3	−0.78 (1.08)	11.6
Wasting	−0.19 (1.1)	4.6	0.18 (1.39)	5.5	−0.01 (1.32)	6.1	0.08 (1.22)	4.1

Key: SD = standard deviation, n = sample size

**Table 5:** Trends in Global and Severe Prevalence of stunting, underweight and wasting in children age 0 to 59 month.

Survey	StuntingGlobal Severe	UnderweightGlobal Severe	WastingGlobal Severe
2002	45.6% 21.6%	27.3% 7.0%	4.6% 1.0%
2007	42.2% 19.5%	13.5% 2.8%	5.5% 2.1%
2013−14	38.1% 16.3%	14.3% 3.1%	6.1% 2.2%
2018	34.7% 11.5%	11.6% 2.1%	4.1% 1.4%

**Table 6:** Prevalence of stunting, underweight and wasting when the data was left-truncated at 6 months (6-59).

Survey	StuntingGlobal Severe	UnderweightGlobal Severe	WastingGlobal Severe
2002	50.4% 24.2%	30.4% 7.9%	4.9% 1.0%
2007	44.1% 20.6%	14.1% 3.0%	5.4% 2.1%
2013−14	40.7% 17.6%	15.3% 3.3%	5.6% 2.1%
2018	36.5% 12.3%	12.5% 2.4%	4.0% 1.3%

**Table 7:** Risk factors extraction for stunting using binary logistic regression.

Variables	Stunting			
	Unadjusted OR (95% CI)	P-value	Adjusted OR (95% CI)	p-value
**Respondent age group** 25-34 ≤ 24 ≥ 35	Ref 1.09 (1.03-1.17)^*^ 0.98 (0.92-1.05)	0.004 0.62	Ref 1.07 (1.00-1.14)^*^ 0.89 (0.83-0.96)^*^	0.04 0.02
**Province** Central C/belt Eastern Luapula Lusaka Muchinga N/Western Northern Southern Western	Ref 0.77 (0.66-0.90)^*^ 1.02 (0.88-1.19) 1.18 (1.00-1.39)^*^ 0.87 (0.75-1.01) 0.99 (0.84-1.18) 0.83 (0.69-1.00) 1.43 (1.25-1.64)^*^ 0.79 (0.69-0.93)^*^ 0.79 (0.67-0.93)^*^	0.001 0.80 0.04 0.07 0.98 0.06 <0.001 0.004 0.004	Ref 0.77 (0.66-0.90)^*^ 1.01 (0.87-1.18) 1.19 (1.01-1.39)^*^ 0.87 (0.74 −1.01) 1.00 (0.84-1.18) 0.83 (0.69-0.99)^*^ 1.44 (1.25-1.65)^*^ 0.79 (0.68-0.93)^*^ 0.78 (0.67 −0.93)^*^	0.001 0.860 0.040 0.060 0.990 0.050 <0.001 0.004 0.004
**Size of child** Average large small	Ref 0.81(0.77-0.87)^*^ 1.51(1.39-1.64)^*^	<0.001 <0.001	Ref 0.81 (0.76-0.86)^*^ 1.49 (1.36-1.62)^*^	<0.001 <0.001
**Wealth Index** Middle Poor Rich	Ref 1.18 (1.09-1.27)^*^ 0.76 (0.69-0.84)^*^	<0.001 <0.001	Ref 1.20 (1.10-1.31)^*^ 0.71 (0.64-0.79)^*^	<0.001 <0.001
**Region** Rural Urban	Ref 0.73(0.77-0.78)^*^	<0.001	Ref 0.84 (0.78-0.90)^*^	<0.001
**Education Level** Tertiary No Education Primary Secondary	Ref 3.59 (2.85-4.52)^*^ 3.48 (2.79-4.35)^*^ 2.50 (1.99-3.15)^*^	<0.001 <0.001 <0.01	Ref 3.11(2.42-3.99)^*^ 3.02(2.38-3.84)^*^ 2.25 (1.77-2.86)^*^	<0.001 <0.001 <0.001
**Gender** Female Male	Ref 1.23 (1.17-1.29)^*^	<0.001	Ref 1.27 (1.21-1.34)^*^	<0.001
**Breast feeding** No Yes	Ref 0.85 (0.80-0.90)^*^	<0.001	Ref 0.77 (0.73-0.81)^*^	<0.001
**Mother’s Working status** No Yes	Ref 1.08 (1.02-1.15)^*^	0.009	Ref 1.08 (1.02-1.15)^*^	0.01
**Toilet facility** Other Pit latrine Water closet	Ref 0.84 (0.78-0.90)^*^ 0.65 (0.58-0.72)^*^	<0.001 <0.001	Ref 0.88 (0.84-0.93)^*^ 0.84 (0.77- 0.92)^*^	<0.001 <0.001
**Water source** Borehole Other Tap Well	Ref 1.11 (0.91-1.34) 0.90 (0.81-1.01)^*^ 1.28 (1.18-1.40)^*^	0.31 0.006 <0.001	Ref 0.89 (0.75-1.05) 0.79 (0.73-0.86)^*^ 1.02 (0.97-1.08)	0.28 0.009 0.08
**Age at first pregnancy**	0.96 (0.95-0.97)^*^	<0.001	0.98 (0.98-0.99)^*^	0.040
**Diarrhea** No Yes	Ref 1.21 (1.13-1.30)^*^	<0.001	Ref 1.19 (1.11, 1.27)^*^	<0.001
**Fever** No Yes	Ref 1.19 (1.11-1.26)^*^	<0.001	Ref 1.12 (1.05, 1.21)^*^	0.001
**Cough** No Yes	Ref 0.99 (0.93-1.05)	0.707	Ref 0.91 (0.85, 0.97)^*^	<0.001

**Table 8:** Risk factors extraction for underweight using binary logistic regression.

Variables	Underweight			
	Unadjusted OR (95% CI)	P-value	Adjusted OR (95% CI)	p-value
**Respondent age group** 25-34 ≤ 24 ≥ 35	Ref 1.07 (0.98-1.16) 1.02 (0.93-1.13)	0.12 0.61	Ref 1.01(0.92-1.10) 0.96 (0.86-1.06)	0.85 0.39
**Province** Central C/belt Eastern Luapula Lusaka Muchinga N/Western Northern Southern Western	Ref (0.80-1.26) 0.84 (0.68-1.04) 1.49 (1.20-1.87) ^*^ 0.81 (0.62-1.05) 1.24 (0.99-1.54) 0.91 (0.71-1.19) 1.36 (1.10-1.67) ^*^ 0.89 (0.69-1.14) 1.21 (0.97-1.54)	0.96 0.11 <0.001 0.12 0.05 0.52 0.004 0.36 0.09	Ref (0.80-1.27) 0.84 (0.67-1.04) 1.51(1.21-1.83) ^*^ 0.82 (0.62-1.06) 1.24 (0.99-1.55) 0.92 (0.71-1.18) 1.36 (1.10-1.68) ^*^ 0.89 (0.69-1.14) 1.22 (0.97-1.54)	0.95 0.10 <0.001 0.126 0.05 0.50 0.004 0.36 0.09
**Size of child** Average large small	Ref 0.67 (0.61-0.73) ^*^ 1.81 (1.71-2.06) ^*^	<0.001 <0.001	Ref 0.66 (0.61-0.72) ^*^ 1.83(1.67-2.01) ^*^	<0.001 <0.001
**Wealth Index** Middle Poor Rich	Ref 1.35 (1.21-1.51) ^*^ 0.84 (0.73-0.97) ^*^	<0.001 0.02	Ref 1.44 (1.29-1.62) ^*^ 0.71 (0.60-0.84) ^*^	<0.001 <0.001
**Region** Rural Urban	Ref 0.78 (0.69-0.870) ^*^	<0.001	Ref 0.94 (0.84-1.06)	0.33
**Education Level** Tertiary No Education Primary Secondary	Ref 3.30 (2.29-4.74) ^*^ 2.62 (1.85-3.73) ^*^ 1.86 (1.30-2.66) ^*^	<0.001 <0.001 0.001	Ref 2.78 (1.89-4.08) ^*^ 2.26 (1.55-3.27) ^*^ 1.65 (1.14-2.40) ^*^	<0.001 <0.001 0.009
**Gender** Female Male	Ref 1.22(1.14-1.31) ^*^	<0.001	Ref 1.28 (1.19-1.38) ^*^	<0.001
**Breast feeding** No Yes	Ref 1.03 (0.95-1.10)	0.49	Ref 0.94 (0.88-1.02)	0.129
**Mother’s Working status** No Yes	Ref 1.08 (1.01-1.17) ^*^	0.04	Ref 1.05 (0.96-1.13)	0.28
**Toilet facility** Other Pit latrine Water closet	Ref 0.60 (0.55-0.66) ^*^ 0.65 (0.57-0.75) ^*^	<0.001 <0.001	Ref 0.88 (0.84,0.93) ^*^ 0.84 (0.77, 0.92) ^*^	<0.001 <0.001
**Water source** Borehole Other Tap Well	Ref 1.24 (0.94-1.64) 1.08 (0.93-1.24) 1.48 (1.34-1.65) ^*^	0.13 0.31 <0.001	Ref 0.89 (0.75-1.05) 0.79 (0.73-0.86) ^*^ 1.02 (0.97-1.08)	0.11 0.35 0.09
**Age at first pregnancy in years**	0.98 (0.96-0.99) ^*^	<0.001	0.99 (0.98-1.01)	0.59
**Diarrhea** No Yes	Ref 1.58 (1.45-1.71) ^*^	<0.001	Ref 1.39 (1.27-1.52) ^*^	<0.001
**Fever** No Yes	Ref 1.61 (1.49-1.75) ^*^	<0.001	Ref 1.45 (1.33-1.58) ^*^	<0.001
**Cough** No Yes	Ref 1.21 (1.12-1.31) ^*^	<0.001	Ref 0.99 (0.91- 1.08)	0.83

**Table 9:** Risk factors extraction for wasting using binary logistic regression.

Variables	Wasting			
	Unadjusted OR (95% CI)	P-value	Adjusted OR (95% CI)	p-value
**Respondent age group** 25-34 ≤ 24 ≥ 35	Ref 1.11 (0.98-1.26) 1.18 (1.01-1.39) ^*^	0.10 0.04	Ref 1.07 (0.94-1.23) 1.21 (1.03-1.43) ^*^	0.32 0.02
**Province** Central C/belt Eastern Luapula Lusaka Muchinga N/Western Northern Southern Western	Ref 1.32 (0.92-1.88) 0.96 (0.66-1.41) 2.50 (1.78-3.52) ^*^ 1.47 (1.02-2.11) ^*^ 1.36 (0.94-1.98) 1.19 (0.83-1.73) 0.74 (0.52-1.07) 0.83 (0.55-1.25) 1.23 (0.86-1.77)	0.13 0.85 <0.001 0.04 0.10 0.35 0.11 0.37 0.26	Ref 1.34 (0.93-1.91) 0.97 (0.67-1.42) 2.53 (1.80-3.54) ^*^ 1.49 (1.04-2.15) ^*^ 1.37 (0.93-1.99) 1.21 (0.83-1.75) 0.74 (0.52-1.07) 0.83 (0.55-1.24) 1.24 (0.86-1.78)	0.11 0.88 <0.001 0.03 0.10 0.32 0.12 0.36 0.25
**Size of child** Average large small	Ref 0.92 (0.79-1.07) 1.44 (1.22-1.70) ^*^	0.29 <0.001	Ref 0.92 (0.79-1.07) 1.43 (1.21-1.68) ^*^	0.26 <0.001
**Wealth Index** Middle Poor Rich	Ref 1.27 (1.06-1.52) ^*^ 1.25 (1.02-1.54) ^*^	0.01 0.03	Ref 1.33 (1.11-1.60) ^*^ 1.14 (0.89-1.44)	0.003 0.29
**Region** Rural Urban	Ref 1.07 (0.93-1.24)	0.32	Ref 1.13 (0.97-1.32)	0.12
**Education Level** Tertiary No Education Primary Secondary	Ref 1.20 (0.82-1.75) 0.96 (0.68-1.37) 1.05 (0.74-1.49)	0.34 0.84 0.79	Ref 1.21 (0.82-1.79) 0.99 (0.69-1.42) 1.07 (0.75-1.53)	0.34 0.96 0.70
**Gender** Female Male	Ref 1.19 (1.05-1.35) ^*^	0.005	Ref 1.21 (1.07-1.37) ^*^	0.002
**Breast feeding** No Yes	Ref 1.30 (1.14-1.48) ^*^	<0.001	Ref 1.31 (1.14-1.49) ^*^	<0.001
**Mother’s Working status** No Yes	Ref 1.01 (0.89-1.14)	0.88	Ref 1.01 (0.89-1.41)	0.91
**Toilet facility** Other Pit latrine Water closet	Ref (0.89-1.18) 1.11 (0.91-1.34)	0.71 0.30	Ref 0.88 (0.84,0.93) ^*^ 0.84 (0.77, 0.92) ^*^	<0.05 <0.05
**Water source** Borehole Other Tap Well	Ref 0.94 (0.61-1.44) 1.11 (0.91-1.37) 1.07 (0.88-1.29)	0.77 0.30 0.49	Ref 0.89 (0.75, 1.05) 0.79 (0.73, 0.86) ^*^ 1.02 (0.97, 1.08)	0.81 <0.05 0.57
**Age at first pregnancy in years**	1.02 (0.99-1.04)	0.16	0.99 (0.98-1.01)	0.59
**Diarrhea** No Yes	Ref 1.51 (1.31-1.75) ^*^	<0.001	Ref 1.44 (1.23-1.67) ^*^	<0.001
**Fever** No Yes	Ref 1.21 (1.06-1.39) ^*^	0.005	Ref 1.16 (0.99-1.35)	0.06
**Cough** No Yes	Ref 1.03 (0.99-1.18)	0.68	Ref 0.91 (0.78-1.06)	0.22

### 2.5. Trend analysis

**Fig 1:**
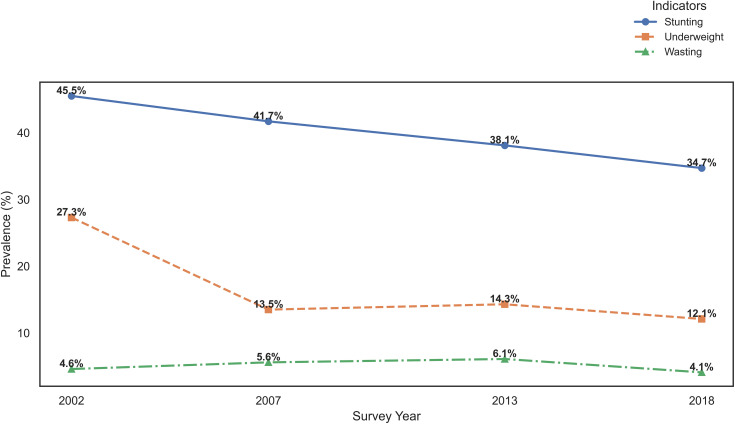
Trends in undernutrition for children under the age of 5 from 2002 to 2018.

Trends in undernutrition among children under five years of age in Zambia were examined using ZDHS data from 2002, 2007, 2013–14, and 2018. Overall trends across the four survey waves were illustrated in Fig 1. In addition, the prevalence of stunting, wasting, and underweight across these four waves, for both global and severe forms of undernutrition, were estimated and presented in Table 5.

**Fig 2:**
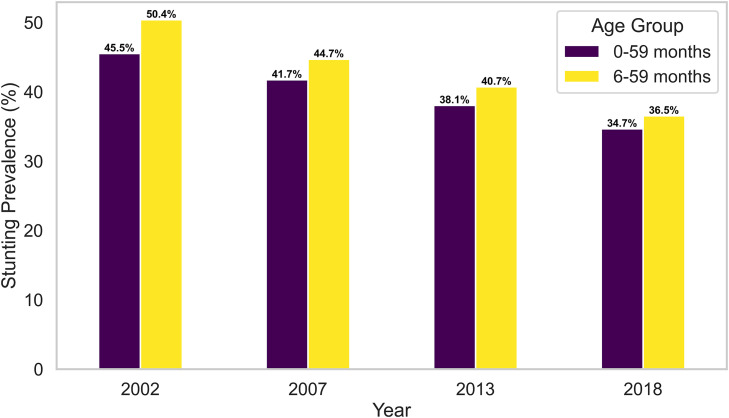
Comparison of stunting prevalence between complete dataset (0 −59 months) verses left-truncated dataset (6–59 months).

**Fig 3:**
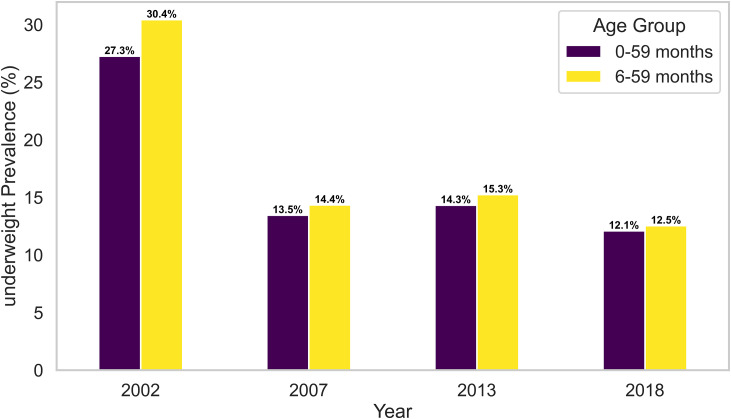
Comparison of underweight prevalence between complete dataset (0-59 months) verses left-truncated dataset (6–59 months).

**Fig 4:**
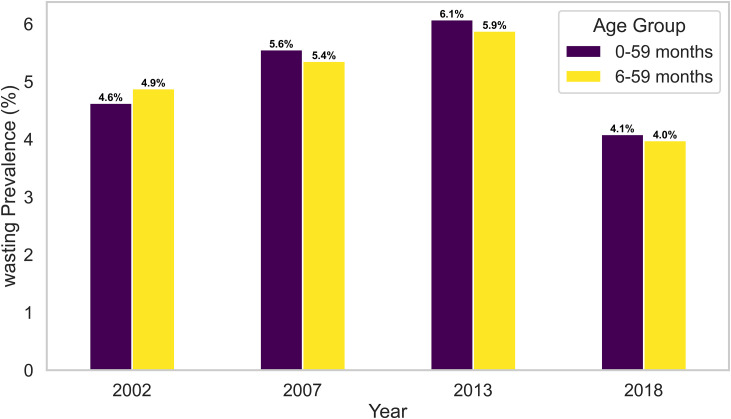
Comparison of wasting prevalence between complete dataset (0 −59 months) verses left-truncated dataset (6–59 months).

To further explore how age-related data completeness influences prevalence estimates, analyses were conducted for two age ranges 0–59 months (using the full data) and 6–59 months (excluding infants younger than six months). The prevalence of stunting, wasting, and underweight for these two age groups is illustrated in Figs 2–4 and summarized in Table 6.

Finally, line graphs and bar charts were generated using Python’s Matplotlib and Seaborn libraries to illustrate patterns.

## 3. Results

This chapter presents a comprehensive analysis of the anthropometric trends in malnutrition among children aged 0–59 months in Zambia. The findings focus on the global and severe prevalence of stunting, underweight, and wasting from 2002 to 2018. The chapter includes visual representations line graphs and bar charts to illustrate these trends. Further analyses explore the impact of left-truncating the dataset at 6 months (i.e., focusing on children aged 6–59 months), demographic and baseline characteristics of the sample population, and multivariate risk factors associated with malnutrition, based on logistic regression models.

### 3.1. Baseline and demographic characteristics of respondents and their associations

Tables 1–3 presents a summary of the baseline and demographic characteristics of survey respondents, using combined data from the 2002, 2007, 2013–2014, and 2018 ZDHS. Due to variable unavailability, the wealth index data were derived only from 2007, 2013, and 2018 datasets, and provincial data were combined from 2013 and 2018, as Muchinga Province was not included in earlier surveys.

Regionally, Northern province exhibited the highest stunting prevalence (45.6%), while Western province recorded the lowest (31.6%), indicating regional disparities in nutritional outcomes. Luapula province had the highest prevalence of wasting at 8.8%, pointing to a higher burden of acute malnutrition, whereas Northern province had the lowest wasting prevalence (3.0%). Underweight prevalence was highest in Luapula (17.4%) and lowest in Eastern province (10.1%). These differences highlight the need for region-specific interventions.

Birth size was strongly associated with malnutrition. Children born small or very small were more likely to be stunted (48.7%), underweight (25.4%), or wasted (6.7%) than those born average or larger in size. Rural residence was also linked to worse outcomes, with stunting rates at 41.4% compared to 34.0% in urban areas. Maternal education was inversely related to stunting; mothers with no education had a stunting prevalence of 42.8%, while those with higher education had only 16.6%. Boys were more affected by stunting (41.4%) than girls (36.7%), suggesting possible gender-related vulnerability.

Several other factors were associated with undernutrition. These include region, residence, mother’s education, household wealth, toilet and water access, breastfeeding practices, and recent illness episodes such as diarrhea. Maternal age at first pregnancy and the sex of the child were also significant predictors of undernutrition. The multifactorial nature of these associations underscores the importance of holistic, integrated approaches to combat malnutrition.

### 3.2. Trends in anthropometric indicators of malnutrition (0–59 months)

The analysis of anthropometric trends among Zambian children aged 0–59 months from 2002 to 2018 reveals notable progress in reducing malnutrition, particularly in stunting and underweight. As shown in Table 4, stunting, measured by the HAZ, has declined from a prevalence of 45.6% in 2002 to 34.7% in 2018. Concurrently, the mean HAZ improved from −1.89 to −1.48, suggesting that, on average, children’s height-for-age has improved significantly over the 17-year period. However, despite these improvements, stunting remains a major public health concern, with over one-third of Zambian children still affected as of 2018 [11].

Underweight, measured by the WAZ, also showed a substantial decline in prevalence, from 27.3% in 2002 to 11.6% in 2018. The mean WAZ increased from −1.31 to −0.78, indicating overall improvement in children’s weight-for-age. The most dramatic reduction occurred between 2001 and 2007, with the prevalence dropping from 27.3% to 13.5%, likely reflecting effective nutrition and health interventions during that period.

Wasting, measured by the WHZ, exhibited relatively stable trends with minor fluctuations. The prevalence ranged from 4.6% in 2002 to 4.1% in 2018. The mean WHZ showed variations over time, increasing to 0.18 in 2007, declining to −0.01 in 2013, and slightly recovering to 0.08 in 2018. This pattern suggests that acute malnutrition has remained relatively stable, with limited progress compared to stunting and underweight.

### 3.3. Visual representation of anthropometric trends (2002–2018)

Fig 1 visually summarizes the overall trends in stunting, underweight, and wasting among children from 2002 to 2018. The line graph shows a steady decline in stunting, from 45.6% in 2002 to 34.7% in 2018, reflecting sustained progress in addressing chronic malnutrition. Underweight prevalence declined sharply between 2002 and 2007, from 27.3% to 13.5%, but the rate of improvement slowed thereafter, reaching 12.1% in 2018. In contrast, wasting displayed a fluctuating trend, with the highest prevalence recorded in 2013 at 6.1%, before decreasing to 4.1% in 2018. These patterns highlight the differing dynamics of chronic versus acute malnutrition, with stunting and underweight showing clearer improvement trajectories.

### 3.4. Global and severe prevalence of stunting, underweight, and wasting

Table 5 provides insight into both global and severe forms of malnutrition from 2002 to 2018. In 2002, the global prevalence of stunting stood at 45.6%, with severe stunting affecting 21.6% of children. By 2018, stunting had declined to 34.7%, while severe stunting dropped more substantially to 11.5%. This indicates meaningful progress in reducing the extreme forms of growth retardation.

The prevalence of underweight children also improved significantly. In 2002, 27.3% of children were underweight, with 7.0% classified as severely underweight. By 2018, the overall prevalence had reduced to 11.6%, and severe underweight dropped to 2.1%, reflecting improved nutritional outcomes and likely better access to food and healthcare.

Wasting followed a less consistent pattern. The global prevalence of wasting was 4.6% in 2002 and declined slightly to 4.1% in 2018. However, severe wasting showed a modest increase from 1.0% in 2002 to 1.4% in 2018. These findings suggest that while acute malnutrition remained relatively low, progress in reducing its severe forms has been limited.

### 3.5. Comparison of prevalences between the complete data (0–59 months) and the left-truncated data (6–59 months)

Table 6 presents anthropometric trends when data are restricted to children aged 6–59 months, allowing for comparison with the full 0–59 months sample. The results from Figs 2–4 reveal that stunting and underweight prevalence were consistently higher among children aged 6–59 months than those aged 0–59 months. In 2002, for example, stunting increased from 45.6% to 50.4%, and underweight rose from 27.3% to 30.4% when children under six months were excluded. This indicates that younger infants typically exhibit lower levels of chronic malnutrition.

Interestingly, the wasting indicator behaved differently. The exclusion of children aged 0–5 months had only a marginal effect on the prevalence of wasting. For instance, in 2007, wasting declined slightly from 5.5% to 5.4% when the data were left-truncated at six months. This minimal change suggests that wasting in infants may be influenced by different factors than stunting and underweight, warranting further investigation.

### 3.6. Logistic regression analysis of risk factors

Tables 7–9 presents the results of a logistic regression analysis on the risk factors for stunting, underweight, and wasting. Significant regional differences are observed. The odds of stunting are 14%, 17%, and 32% lower for children in Northwestern, Southern, and Western provinces, respectively, compared to Central province. Conversely, the odds of underweight are 14% and 28% higher in Luapula and Northern provinces, respectively. For underweight, children in Eastern province have a 28% lower risk, while those in Copperbelt and Luapula provinces have 24% and 36% higher odds, respectively. With reference to wasting, children in Northern province have a 35% lower risk, whereas those in Luapula and Lusaka provinces are twice as likely and 40% more likely to be wasted, respectively, compared to Central province.

Birth size has a significant association with all three forms of undernutrition. Children born small or very small have 45% higher odds of stunting and 75% higher odds of underweight compared to those born average or large. Wealth status also plays a role, as children from poor households have a 30% higher likelihood of being stunted than those from middle-income households.

Maternal education was a strong predictor of nutritional status. Children whose mothers had no formal education were 2.83 times more likely to be stunted and 2.43 times more likely to be underweight than those with mothers who had higher education. Male children were disproportionately affected, with boys having 26% higher odds of stunting, 25% higher odds of underweight, and 22% higher odds of wasting compared to girls.

Breastfeeding offered a protective effect against stunting, reducing its odds by 22%, although it did not significantly affect wasting. Children of working mothers had a 7% higher risk of stunting. Improved sanitation and water sources were associated with reduced undernutrition, suggesting that environmental health plays a key role.

Recent illnesses, particularly diarrhea and fever, were significantly linked to malnutrition. Children who had diarrhea in the past two weeks were 17% more likely to be stunted and 37% more likely to be underweight. Fever increased the odds of stunting by 8% and underweight by 34%, although it did not significantly influence wasting. These findings highlight the importance of infection control in reducing childhood malnutrition.

Table 7 presents the unadjusted and adjusted odds ratios for factors associated with stunting among children under the age of 5 years. After controlling for potential confounders, several demographic, socioeconomic, environmental, and health-related variables remained significantly associated with stunting.

**Maternal age:** Children born to mothers aged ≤24 years had significantly higher odds of being stunted compared to those whose mothers were aged 25–34 years (AOR = 1.07; 95% CI: 1.00–1.14). In contrast, children of mothers aged ≥35 years were significantly less likely to be stunted (AOR = 0.89; 95% CI: 0.83–0.96), suggesting a protective effect of older maternal age Table 7.

**Province of residence:** Significant provincial disparities in stunting were observed. Compared to Central Province, children residing in Eastern (AOR = 1.19; 95% CI: 1.01–1.39) and Northern provinces (AOR = 1.44; 95% CI: 1.25–1.65) had significantly higher odds of stunting. Conversely, residence in Copperbelt, Muchinga, Southern, and Western provinces was associated with lower odds of stunting. These findings highlight pronounced geographic inequalities in chronic child undernutrition Table 7.

**Size of child at birth:** Child size at birth was a strong predictor of stunting. Children perceived as small at birth had significantly higher odds of being stunted (AOR = 1.49; 95% CI: 1.36–1.62), while those perceived as large at birth were less likely to be stunted (AOR = 0.81; 95% CI: 0.76–0.86), underscoring the long-term nutritional consequences of intrauterine growth restriction Table 7.

**Household wealth:** Household socioeconomic status was strongly associated with stunting. Children from poor households had significantly higher odds of stunting compared to those from middle-income households (AOR = 1.20; 95% CI: 1.10–1.31), whereas children from rich households were significantly less likely to be stunted (AOR = 0.71; 95% CI: 0.64–0.79).

**Place of residence:** Children living in urban areas had lower odds of stunting compared to their rural counterparts (AOR = 0.84; 95% CI: 0.78–0.90), reflecting better access to health services, sanitation, and food resources in urban settings.

**Maternal education:** Maternal education emerged as one of the strongest predictors of stunting. Compared to mothers with tertiary education, children whose mothers had no education (AOR = 3.11; 95% CI: 2.42–3.99), primary education (AOR = 3.02; 95% CI: 2.38–3.84), or secondary education (AOR = 2.25; 95% CI: 1.77–2.86) were significantly more likely to be stunted, indicating a strong educational gradient.

**Child’s sex:** Male children had significantly higher odds of stunting compared to female children (AOR = 1.27; 95% CI: 1.21–1.34), suggesting greater biological vulnerability or gender-related differences in care.

**Breastfeeding status:** Breastfeeding was significantly protective against stunting. Breastfed children had lower odds of stunting compared to those who were not breastfed (AOR = 0.77; 95% CI: 0.73–0.81).

**Maternal employment:** Children of working mothers were slightly more likely to be stunted than those whose mothers were not working (AOR = 1.08; 95% CI: 1.02–1.15), possibly reflecting reduced caregiving time or reliance on suboptimal childcare arrangements.

**Environmental health factors:** Access to improved sanitation significantly reduced the likelihood of stunting. Children from households with pit latrines (AOR = 0.88; 95% CI: 0.84–0.93) or water closets (AOR = 0.84; 95% CI: 0.77–0.92) were less likely to be stunted compared to those using unimproved toilet facilities. Improved water sources also showed a protective effect, although associations weakened after adjustment.

**Maternal reproductive history:** Increasing age at first pregnancy was associated with lower odds of stunting (AOR = 0.98; 95% CI: 0.98–0.99), suggesting that delayed childbearing may improve child nutritional outcomes.

**Child morbidity:** Recent episodes of diarrhea (AOR = 1.19; 95% CI: 1.11–1.27) and fever (AOR = 1.12; 95% CI: 1.05–1.21) were significantly associated with higher odds of stunting, highlighting the role of repeated infections in impairing linear growth. Cough was not significant in the unadjusted analysis but showed a modest protective association after adjustment.

Table 8 presents the unadjusted and adjusted odds ratios for factors associated with underweight among children under the age of 5. After adjustment for potential confounders, several socioeconomic, biological, environmental, and health-related factors remained significantly associated with underweight.

**Maternal age:** Maternal age was not significantly associated with underweight in either the unadjusted or adjusted models. Children of mothers aged ≤24 years (AOR = 1.01; 95% CI: 0.92–1.10) and ≥35 years (AOR = 0.96; 95% CI: 0.86–1.06) did not differ significantly in their likelihood of being underweight compared to those whose mothers were aged 25–34 years Table 8.

**Province of residence:** Marked provincial differences in underweight prevalence were observed. After adjustment, children residing in Eastern Province had significantly higher odds of being underweight (AOR = 1.51; 95% CI: 1.21–1.83), as did those living in Northern Province (AOR = 1.36; 95% CI: 1.10–1.68), compared to Central Province. No statistically significant differences were observed for the remaining provinces Table 8.

**Size of child at birth:** Size at birth was a strong determinant of underweight. Children perceived as small at birth were nearly twice as likely to be underweight (AOR = 1.83; 95% CI: 1.67–2.01), whereas those perceived as large at birth had significantly lower odds of underweight (AOR = 0.66; 95% CI: 0.61–0.72). This highlights the lasting impact of fetal growth on postnatal nutritional status.

**Household wealth:** Household wealth status was significantly associated with underweight. Children from poor households had increased odds of being underweight (AOR = 1.44; 95% CI: 1.29–1.62), while those from rich households were significantly less likely to be underweight (AOR = 0.71; 95% CI: 0.60–0.84), compared to children from middle-income households.

**Place of residence:** Although rural residence was associated with underweight in the unadjusted analysis, this association was no longer statistically significant after adjustment (AOR = 0.94; 95% CI: 0.84–1.06), suggesting that the rural–urban difference is largely explained by other socioeconomic and environmental factors.

**Maternal education:** Maternal education showed a strong and graded association with underweight. Compared to mothers with tertiary education, children whose mothers had no education (AOR = 2.78; 95% CI: 1.89–4.08), primary education (AOR = 2.26; 95% CI: 1.55–3.27), or secondary education (AOR = 1.65; 95% CI: 1.14–2.40) were significantly more likely to be underweight.

**Child’s sex:** Male children had significantly higher odds of being underweight compared to female children (AOR = 1.28; 95% CI: 1.19–1.38), consistent with observed sex-based differences in child nutritional vulnerability.

**Breastfeeding status:** Breastfeeding status was not significantly associated with underweight after adjustment (AOR = 0.94; 95% CI: 0.88–1.02), indicating no independent effect once other factors were controlled for.

**Maternal employment:** While maternal employment was associated with underweight in the unadjusted model, this association did not persist after adjustment (AOR = 1.05; 95% CI: 0.96–1.13), suggesting confounding by socioeconomic or household factors.

**Environmental health factors:** Access to improved sanitation was significantly protective against underweight. Children from households using pit latrines (AOR = 0.88; 95% CI: 0.84–0.93) or water closets (AOR = 0.84; 95% CI: 0.77–0.92) were less likely to be underweight compared to those using unimproved toilet facilities. Improved water sources showed limited independent effects after adjustment.

**Maternal reproductive history:** Maternal age at first pregnancy was not significantly associated with underweight in the adjusted model (AOR = 0.99; 95% CI: 0.98–1.01), despite showing a modest protective effect in the unadjusted analysis.

**Child morbidity:** Recent illness was a strong predictor of underweight. Children who had experienced **diarrhea** (AOR = 1.39; 95% CI: 1.27–1.52) or **fever** (AOR = 1.45; 95% CI: 1.33–1.58) had significantly higher odds of being underweight. Cough was significant only in the unadjusted analysis and lost significance after adjustment.

Table 9 presents the unadjusted and adjusted odds ratios for factors associated with wasting among children under the age of 5 years. Unlike stunting and underweight, wasting an indicator of acute malnutrition was primarily associated with maternal age, selected provincial effects, child-level biological factors, sanitation, and recent morbidity.

**Maternal age:** Maternal age showed a modest but significant association with wasting. After adjustment, children of mothers aged ≥35 years had significantly higher odds of wasting compared to those whose mothers were aged 25–34 years (AOR = 1.21; 95% CI: 1.03–1.43). No significant association was observed for mothers aged ≤24 years Table 9.

**Province of residence:** Pronounced regional disparities were observed for wasting. Compared to Central Province, children residing in Eastern Province had more than twice the odds of being wasted (AOR = 2.53; 95% CI: 1.80–3.54), while those in Luapula Province also had significantly higher odds (AOR = 1.49; 95% CI: 1.04–2.15). No statistically significant differences were observed for the remaining provinces after adjustment, highlighting localized vulnerability to acute malnutrition.

**Size of child at birth:** Size at birth was a significant predictor of wasting. Children perceived as small at birth had higher odds of wasting (AOR = 1.43; 95% CI: 1.21–1.68), whereas perceived large size at birth was not significantly associated with wasting. This finding suggests that poor fetal growth increases susceptibility to acute nutritional deficits later in childhood.

**Household wealth:** Household wealth showed a weaker association with wasting compared to stunting and underweight. Children from poor households had higher odds of wasting in the unadjusted model; however, this association was no longer statistically significant after adjustment (AOR = 1.14; 95% CI: 0.89–1.44). Children from middle**-**income households, however, remained at increased risk (AOR = 1.33; 95% CI: 1.11–1.60), suggesting that acute malnutrition may be influenced by transient shocks affecting broader segments of the population.

**Place of residence:** Place of residence was not significantly associated with wasting in either the unadjusted or adjusted models, indicating that acute malnutrition affects children in both rural and urban settings.

**Maternal education:** Maternal education was not significantly associated with wasting after adjustment. This contrasts with the strong educational gradients observed for stunting and underweight, reinforcing the notion that wasting is driven more by immediate factors than long-term socioeconomic conditions.

**Child’s sex:** Male children were significantly more likely to be wasted than female children (AOR = 1.21; 95% CI: 1.07–1.37), consistent with observed sex-based vulnerability in acute malnutrition.

**Breastfeeding status:** Breastfeeding was positively associated with wasting. Breastfed children had higher odds of wasting compared to non-breastfed children (AOR = 1.31; 95% CI: 1.14–1.49). This likely reflects reverse causality, whereby mothers increase or maintain breastfeeding in response to child illness or weight loss rather than breastfeeding causing wasting.

**Maternal employment:** Maternal working status was not significantly associated with wasting in either the unadjusted or adjusted analyses.

**Environmental health factors:** Improved sanitation was significantly protective against wasting. Children from households with pit latrines (AOR = 0.88; 95% CI: 0.84–0.93) or water closets (AOR = 0.84; 95% CI: 0.77–0.92) had lower odds of wasting compared to those using unimproved facilities. Improved water sources showed limited independent effects after adjustment.

**Maternal reproductive history:** Age at first pregnancy was not significantly associated with wasting in either the unadjusted or adjusted models.

**Child morbidity:** Recent illness was a key determinant of wasting. Children who experienced diarrhea had significantly higher odds of wasting (AOR = 1.44; 95% CI: 1.23–1.67). Fever showed a positive association in the unadjusted model but lost statistical significance after adjustment, while cough was not associated with wasting.

## 4. Discussion

The findings of this study provide valuable insights into the trends and determinants of undernutrition among children under five in Zambia between 2002 and 2018. The results indicate significant progress in reducing stunting and underweight, while wasting has remained relatively unchanged. This pattern suggests that while long-term interventions may be mitigating chronic malnutrition, acute malnutrition persists reflecting gaps in short-term food security and child healthcare. Similar trends have been observed in other low- and middle-income countries, such as Nigeria and Ethiopia, where stunting has declined but wasting remains prevalent [9,6].

The decline in stunting from 45.6% in 2002 to 34.7% in 2018 reflects improvements in child nutrition, healthcare access, and socio-economic conditions. However, stunting remains a significant concern, still affecting more than one-third of Zambian children. These findings are consistent with national-level studies, such as that by [12], which highlighted the role of food insecurity, poor maternal health, and inadequate feeding practices. Internationally, similar associations have been documented in South Asia and sub-Saharan Africa, where maternal undernutrition and low dietary diversity are key drivers of child stunting [2,13].

The prevalence of underweight declined markedly from 27.3% in 2002 to 11.6% in 2018, with the most rapid improvements observed between 2002 and 2007. This aligns with Lusaka-based research associating improvements in maternal nutritional knowledge and feeding practices with reduced child underweight [14]. However, the slower decline after 2007 suggests need for sustained investment in child nutrition programs and maternal support systems. A similar plateauing effect was observed in Kenya and Uganda, where initial nutrition programs achieved early gains that were not maintained due to funding and implementation gaps [15,16].

In contrast, wasting prevalence has fluctuated slightly, from 4.6% in 2002 to 4.1% in 2018, while severe wasting increased from 1.0% to 1.4%. This pattern reflects the episodic nature of acute malnutrition, influenced by short-term factors like disease outbreaks, seasonal food insecurity, and natural disasters. Similar findings have been reported in West African countries, where wasting rates remain stubbornly high due to climate shocks and limited access to emergency nutrition services [9].

This study also reveals significant regional disparities in nutritional outcomes. Northern Province recorded the highest stunting rate (45.6%), while Western Province had the lowest (31.6%). Luapula Province had the highest rates of both wasting (8.8%) and underweight (17.4%). These findings are consistent with findings from Malawi and Ethiopia where rural, impoverished regions face higher undernutrition burdens [6].

Maternal education emerged as a strong protective factor. Children whose mothers had no formal education were 2.83 times more likely to be stunted than those whose mothers attained higher education Table 7. This mirrors findings from studies in Nigeria and Bangladesh, which confirm that maternal education significantly reduces the risk of child undernutrition through improved health-seeking behavior and better child feeding practices [9,17]. Lusaka-based research further supports this, showing that poor maternal understanding of malnutrition prevention contributes to elevated child undernutrition rates [14].

The logistic regression analysis confirms that multiple socio-economic and environmental factors significantly contribute to undernutrition. Birth size was a strong predictor, children born small or very small had higher odds of being stunted or underweight Tables 7 and 8. This is consistent with findings from India and Nepal, where low birth weight is a major risk factor for poor nutritional outcomes [2]. These findings underscore the importance of maternal nutrition and antenatal care in reducing neonatal and infant health risks.

Household wealth also played a critical role. Children from poor households were 30% more likely to be stunted than those from middle-income households. Similar findings have been reported in multi-country analyses across sub-Saharan Africa and South Asia [9]. Targeted poverty-reduction strategies such as cash transfers and food supplementation are essential to address these disparities.

Access to water, sanitation, and hygiene (WASH) was another important determinant. Children in households with access to improved toilet facilities and clean water were less likely to be malnourished. These results are supported by regional studies from Ethiopia and Ghana, which found that improved WASH conditions significantly reduced child undernutrition.

Other consistent risk factors include frequent childhood illnesses, such as diarrhea and fever, which were associated with higher rates of stunting and underweight. Repeated infections increase metabolic demands and reduce nutrient absorption, exacerbating malnutrition [18]. Similar associations have been reported in both local [14] and international studies [2]. Strengthening healthcare systems, expanding immunization coverage, and improving caregiver education on disease prevention are crucial to breaking the infection-malnutrition cycle.

### 4.1. Strengths and Limitations

This study has several notable strengths. First, it uses nationally representative ZDHS data collected over nearly two decades, enabling a robust assessment of temporal trends in undernutrition among children under five in Zambia. The application of standardized anthropometric indicators and multivariate logistic regression strengthens the internal validity of the findings and supports comparisons across different regions and time points.

Second, the study uniquely compares undernutrition prevalence based on both complete data (0–59 months) and left-truncated data (6–59 months). This distinction is important because it highlights how exclusion of younger infants who tend to have lower prevalence of stunting and underweight can inflate overall estimates. Such methodological transparency improves the interpretability of findings and has implications for survey design and policy interpretation.

Third, the analysis includes both overall and **severe forms** of stunting, wasting, and underweight, which allows for a more nuanced understanding of the severity of child undernutrition. This approach enables better alignment with global targets (e.g., WHO thresholds) and enhances the utility of the results for international benchmarking and prioritization of high-risk cases.

However, the study is not without limitations. Although the original aim was to analyze nutritional trends from 1992 to 2024, this was not feasible due to incomplete or unavailable data for the 1992, 1997, and 2024 surveys. Consequently, the analysis was limited to data from 2001 to 2018, potentially missing earlier and more recent developments.

Additionally, the cross-sectional nature of the ZDHS limits the ability to draw causal inferences. Several important determinants of child nutritional status such as dietary diversity, household food insecurity, and comprehensive measures of maternal nutritional status were either not collected, inconsistently available, or omitted from the surveys, limiting the ability to fully assess their influence. The absence of these variables may contribute to residual confounding, particularly given their well-established roles in shaping child growth outcomes. Furthermore, self-reported measures such as child illness and birth size may be subject to recall and reporting biases. Finally, unobserved factors, including intra-household food allocation, maternal mental health, and caregiving practices, were not captured in the dataset and may also influence nutritional outcomes.

### 4.2. Policy Implications

The findings have several policy implications for Zambia’s ongoing efforts to reduce child undernutrition:

- Strengthen maternal education programs by integrating nutrition education and health literacy into both formal and informal settings.
- Expand social protection measures, such as targeted cash transfers and food support, especially for rural and low-income households.
- Enhance WASH infrastructure to reduce infection-related malnutrition, particularly in underserved regions.
- Invest in maternal and child healthcare, including prenatal care and community-based growth monitoring, to prevent low birth weight and promote early detection of undernutrition.
- Ensure sustainable funding for national nutrition programs to avoid stagnation in progress, especially for reducing wasting.

### 4.3. Future research directions

Future studies should explore the following areas:

- Longitudinal studies to assess causal pathways and the long-term impact of early-life undernutrition.
- Qualitative research to understand community-level barriers to maternal and child nutrition.
- Evaluation of specific interventions, such as community-based nutrition programs or emergency feeding schemes, to identify the most effective strategies.
- Analysis of emerging trends, such as urban undernutrition or the impact of climate change on child health, using more recent ZDHS or other national survey data.

## 5. Conclusion

Although Zambia has made commendable progress in reducing stunting and underweight, undernutrition remains a pressing public health concern. The persistent regional and socio-economic disparities highlight the need for targeted interventions that address localized challenges. Comparisons with previous studies reinforce that multiple factors including maternal education, household wealth, healthcare access, and sanitation play significant roles in child nutritional outcomes. A comprehensive, multi-sectoral approach that integrates nutrition, healthcare, education, and social protection is essential to achieving sustainable improvements in child nutrition and ensuring that all Zambian children have a healthy start in life.

### 5.1 What is already known on this topic

Several studies have analyzed the prevalence of stunting, wasting, and underweight at each data points, consistently highlighting that malnutrition among children under five remains a major public health concern.

It is also well established in the literature that the burden of malnutrition is not evenly distributed across countries or regions. In Zambia, national surveys like the ZDHS have consistently shown high levels of stunting, particularly in rural provinces such as Northern and Luapula, pointing to persistent regional inequalities in nutritional outcomes. For instance, [2] and UNICEF reports have emphasized that socioeconomic factors, maternal education, and access to health services contribute significantly to these disparities.

Additionally, most previous studies on child nutrition in Zambia and similar settings have used the full sample of children aged 0–59 months when reporting prevalence rates. Few have explored how prevalence estimates might shift if different age sub-groups such as excluding children under six months.

### 5.2 What this study adds

This study adds new insights into how the choice of age group influences the measurement and interpretation of child malnutrition in Zambia. By comparing the full dataset (children aged 0–59 months) with a truncated dataset (children aged 6–59 months), we reveal that excluding the youngest infants who are often exclusively breastfed and less exposed to inadequate complementary feeding leads to higher prevalence estimates for both global and severe forms of stunting and underweight. This pattern was consistent across all four DHS survey years (2002, 2007, 2013/14, and 2018), highlighting that age-based inclusion criteria can significantly affect the perceived burden of malnutrition.

In contrast, wasting showed a slight decrease in the truncated sample, while severe wasting increased marginally, illustrating the different age-related patterns of acute versus chronic malnutrition. By disaggregating both global and severe forms of each indicator, our study provides a more nuanced understanding of nutritional trends, showing that while overall levels of severe stunting dropped from 21.6% to 11.5% over the study period, severe forms still represent a substantial public health concern.

Additionally, our study confirm that boys are consistently more affected than girls across all three indicators offering robust local evidence that supports global patterns and strengthens the case for gender-sensitive nutrition interventions in Zambia. Overall, this study emphasizes that methodological decisions especially regarding age stratification and the inclusion of severity levels can shape not only prevalence estimates but also policy priorities.

Our study informs policymakers by providing a more detailed understanding of childhood malnutrition in Zambia through regional and trend analysis, as well as by highlighting the importance of disaggregating severity levels in stunting, wasting, and underweight. By demonstrating that excluding children under six months yields higher prevalence estimates particularly for stunting and underweight we show how methodological decisions can significantly affect the interpretation of nutritional data. The consistent finding that boys are more affected than girls also strengthens the case for gender-sensitive nutrition interventions. These insights support the need for more targeted, age-specific, and regionally responsive policies to effectively address both chronic and acute forms of child undernutrition.
